# Slaughtering of Water Buffalo (*Bubalus bubalis*) with and without Stunning: A Focus on the Neurobiology of Pain, Hyperalgesia, and Sensitization

**DOI:** 10.3390/ani13152406

**Published:** 2023-07-25

**Authors:** Temple Grandin, Antonio Velarde, Ana Strappini, Marien Gerritzen, Marcelo Ghezzi, Julio Martínez-Burnes, Ismael Hernández-Ávalos, Adriana Domínguez-Oliva, Alejandro Casas-Alvarado, Daniel Mota-Rojas

**Affiliations:** 1 Department of Animal Science, Colorado State University, Fort Collins, CO 80526, USA; cheryl.miller@colostate.edu; 2 Animal Welfare Program, Institute of Agrifood Research and Technology (IRTA), Veinat Sies S-N, 17121 Monells, Spain; antonio.velarde@irta.cat; 3 Animal Health & Welfare, Wageningen Livestock Research, Wageningen University & Research, 6708 WD Wageningen, The Netherlands; ana.strappini@wur.nl (A.S.); marien.gerritzen@wur.nl (M.G.); 4 Animal Welfare Area, Faculty of Veterinary Sciences (FCV), Universidad Nacional del Centro de la Provincia de Buenos Aires (UNCPBA), University Campus, Tandil 7000, Argentina; ghezzi@vet.unicen.edu.ar; 5 Facultad de Medicina Veterinaria y Zootecnia, Universidad Autónoma de Tamaulipas, Victoria City 87000, Mexico; 6 Clinical Pharmacology and Veterinary Anesthesia, FESC, Universidad Nacional Autónoma de México (UNAM), Cuautitlán Izcalli 54714, Mexico; 7 Neurophysiology, Behavior and Animal Welfare Assessment, DPAA, Universidad Autónoma Metropolitana (UAM), Mexico City 04960, Mexico

**Keywords:** nociception, consciousness, stunning, *Bubalus bubalis*, animal welfare

## Abstract

**Simple Summary:**

Due to anatomical differences in the skull of the water buffalo (*Bubalus bubalis*), the currently applied mechanical stunning methods in cattle (genus *Bos*) have shown to be ineffective in buffaloes by not damaging vital cerebral structures associated with consciousness. The present review analyzes these anatomical particularities of water buffaloes to discuss the importance of selecting a stunning method suitable for the species to avoid pain and suffering. Potential pain-related processes such as hyperalgesia and sensitization are also addressed. In addition, the signs to assess stun quality and return to consciousness are discussed to elucidate the importance of recognizing these indicators and prevent physiological consequences and affectation to water buffaloes’ welfare and quality of death.

**Abstract:**

The slaughter process in livestock is considered a stressor where the transport and handling of animals, as well as the selected stunning and bleeding methods, can cause acute pain, distress, and suffering. In water buffaloes, although stunning is known to be performed before bleeding to induce unconsciousness, no emphasis is made on the nociceptive events during this process. Particularly, current mechanical stunning methods applied to cattle are unsuitable for water buffaloes due to anatomical differences in the skull from other large ruminants. Furthermore, although very high-pressure pneumatic (200–220 psi) may be effective in the frontal position for lighter-weight water buffalos, for heavier animals, it is less likely to be effective. The present review aims: (1) to analyze the anatomical particularities of water buffaloes to discuss the importance of selecting a stunning method suitable for buffaloes, and (2) to revise the potential pain-related consequences, such as hyperalgesia and sensitization, and the signs to assess the stun quality and death to comprehend the relevance of a proper technique according to the species.

## 1. Introduction

Stunning is performed in livestock destined for human consumption to induce unconsciousness and prevent pain perception. Stunning techniques for cattle (*Bos indicus*, *Bos taurus*) are widely acknowledged in several worldwide official guidelines. However, in the case of the water buffalo (*Bubalus bubalis*), a domestic species whose productive meat value is increasing, the stunning process is not always effective. The latter may be due to the anatomical characteristics of the water buffalo (i.e., hard bone plates, thick hide, profound and extensive frontal sinuses), together with the horn and head size that might influence the slaughter position [1], which cause the failure of conventional penetrative captive bolt techniques and are therefore not able to induce unconsciousness [2,3,4]. Penetrating captive bolt is the most used stunning method for water buffaloes. This is due to the ejected force that can penetrate osseous structures and cause unconsciousness. The lack of a specialized stunning technique for water buffaloes raises welfare concerns about the slaughter process in the species. As it is inefficient, the animal may experience acute pain due to nociception during the stunning and slaughter, affecting not only the welfare of the slaughtered animal, but also the quality of the meat [2,5,6,7,8,9]. In this sense, water buffalo meat is considered a high-quality and high-nutritional product, requiring adequate stunning methods to maintain meat value [10].

An inefficient stunning can arise due to technical limitations or a lack of knowledge and training of the stock person on the species. Moreover, restraint positions and devices can also affect the quality of the stunning by applying excessive pressure or mispositioning the animal, preventing damage to fundamental cerebral structures and affecting their welfare [11]. Both scenarios can induce nociception, a process triggered by recognizing a potential noxious stimulus when the animals are conscious [2,12,13,14].

The potential relationship between the degree of nociception and sensitization during the slaughter process has yet to be extensively determined in water buffaloes [4,15,16]. The present review has two aims: (1) to analyze the anatomical particularities that must be considered to select an appropriate stunning method for water buffaloes and (2) to discuss the importance of the different stunning methods to induce unconsciousness and prevent possible pain-related consequences such as hyperalgesia and sensitization. The signs currently used to assess the quality of stunning and death in water buffaloes are also described.

## 2. Anatomical Features of the Water Buffalo Skull

The water buffalo is a species similar to the domestic bovine of the genus Bos, but critical anatomical differences have been reported in buffaloes [17,18]. In the water buffalo, the mean distance from the skin surface to the inner face (facies interna) of the frontal bone (os frontal) is larger than in cattle (74.0 mm ± 22 vs. 36.0 mm 6 ± 7.5) and also from the skin to the thalamus (144.8 mm ± 27.5 vs. 102.0 mm ± 10.5, respectively) [19].

**Figure 1:**
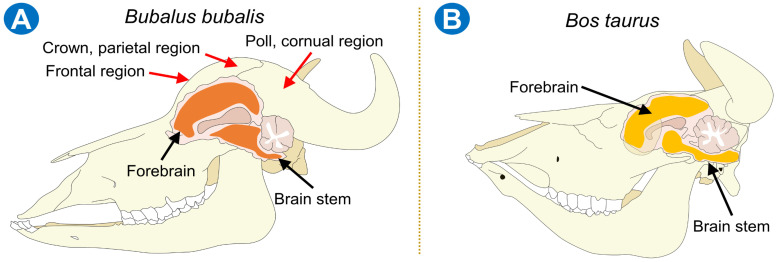
Anatomical comparison of skull osseous structure in cattle (*Bos taurus*) and water buffalo (*Bubalus bubalis*). (**A**) To avoid an inadequate stunning technique, positioning the device in the frontal region, crown or parietal region, and poll or cornual region is recommended for inducing unconsciousness in water buffaloes due to forebrain and brainstem damage. However, the length of the bolt also influences the deepness of the damage. (**B**) Osseous and brain differences in *Bos taurus*.

The difference in bone thickness was studied by Özkan et al. [20], who obtained the craniometric measurements through a morphometric analysis in 15 water buffaloes and 20 bovines (*Bos taurus*). The authors observed that the least frontal breadth was 17% higher in water buffaloes than in conventional cattle. Additionally, the least and greatest heights of the occipital region were 28% and 4% higher in buffalo versus *Bos* cattle, respectively. Likewise, Alsafy et al. [1] compared the skull of the Egyptian buffalo (*Bubalus bubalis*) and cattle (*Bos taurus*) using computed tomography. The results showed that, in buffaloes, the nasal septum reaches the floor of the nasal cavity caudally, and there is a vomeronasal organ at each side of the nasal septum, unlike cattle, where the septum does not reach the nasal cavity, and the nasal cavity forms a medial canal that continues into the nasopharynx. Thus, this suggests that water buffaloes have more profound and extensive frontal sinuses within the frontal and parietal, bones located on the rostro–dorsal and lateral sides of the head, respectively, in addition to a highly developed middle interfrontal septum, thicker skin (epidermis thickness 50–115 µm vs. 51 µm when compared to *Bos taurus*), and a harder bone plate (74 mm vs. 36.6 mm, respectively) (Figure 1) [1,2,3,21,22].

Understanding these differences make it comprehensible why mechanical stunning methods applied in *Bos* cattle are ineffective in water buffaloes, leading to severe pain. In this sense, Gregory et al. [23] evaluated the prevalence of shallow depth concussion following penetrative captive bolts powered by cartridge, compressed air, and firing in 1608 heavy cattle (over 480 kg). A prevalence of 8% of shallow concussion was reported for all cattle, specifying that soft-sounding shots (≤111 dB) and using 4.5 g cartridges cause little shallow depth of concussion. This suggests that in water buffaloes, conventional penetrative stunning methods powered by cartridges performed at the frontal region (*Regio frontalis*) are not inducing unconsciousness in all animals due to anatomical characteristics such as the deep and hard bone tissue in the frontal region. In contrast, compressed air guns have enough force to penetrate the thickness of this bone tissue [24]. Oliveira et al. [25] assessed 304 purebred zebu animals and 139 cross-bred cattle to compare two methods of stunning with low-air-pressure (160–175 psi) and high-pressure (190 psi) pistols. They observed that animals shot with low pressure had a 21% higher incidence of rhythmic respirations, 8% less protrusion of the tongue, and 26% less relaxation of the masseter muscles than animals that were shot with high-pressure guns. Therefore, this is an example of how compressed air guns could be used for water buffaloes. Furthermore, the length of conventional captive bolt devices is 9 cm. Schwenk et al. [19] reported that applying penetrating bolts in a poll position with a protruding length of 12 cm might provide an effective stun in water buffaloes [19].

Gregory et al. [4] evaluated the efficacy of captive bolt pistol shooting in the frontal (*Regio frontalis*), crown in parietal (*Regio parietalis*), and poll in the occipital (*Regio occipitalis*) regions in 30 water buffaloes to address this issue. They found that shooting in the frontal region was ineffective since collapse was not achieved. In the crown, the animals resumed breathing shortly after collapsing, indicating a superficial depth with a prevalence of 53%. In contrast, the poll region showed a greater effectiveness in stunning with all the animals collapsing. According to these results, the poll region could be an option for water buffalo stunning to confirm that animals are unconscious before slaughter, aiming to prevent the nociceptive consequences of inadequate stunning application.

## 3. Importance of Stunning and Nociception before Slaughter

Livestock slaughtering can be painful due to exposure to noxious stimuli that peripheral fibers will recognize and transmit to central brain structures [26,27,28]. Inducing unconsciousness is critical to preventing pain perception by inactivating the cerebral structures involved in pain pathways (e.g., thalamus, cerebral cortex, amygdala, locus coeruleus, hippocampus, and hypothalamus) [29]. Pain causes the release of proinflammatory mediators such as prostaglandins (PG), prostacyclin (PC), serotonin (5-HT), histamine, bradykinin (BK), and leukotrienes. These substances sensitize peripheral receptors that recognize noxious inputs (nociceptive fibers). The persistent activation of nociceptors when animals are not stunned correctly can cause the development of hyperalgesia and peripheral or central sensitization [15,30,31]. It is necessary to discuss this phenomenon in the following paragraphs to understand the consequences that nociception would have during slaughter.

### 3.1. Slaughter with and without Stunning and Its Nociceptive Effects

Pain is an implicit event that can occur during slaughtering when no appropriate stunning is performed or when it does not induce an immediate loss of consciousness [32]. Bleeding/sticking facilitates the loss of consciousness and death due to hypovolemic shock [13,33]. Without stunning, livestock can perceive pain, and it is described that animals can experience pain for at least 60 s before unconsciousness occurs [34]. Studies evaluating neurophysiological parameters have shown that most cattle lose consciousness between 5 and 90 s after the cut. However, the resurgence of consciousness has been reported for more than 5 min [35]. Studies during slaughtering are limited in water buffaloes, but a similar reaction could be expected. Therefore, apart from the stress it represents, the slaughter must be performed with as little pain as possible, which can only be achieved by applying effective stunning methods.

Soft tissue cutting of the neck is one of the events triggering the generation of nerve impulses due to noxious signals perceived by nociceptors in muscles, skin, and viscera [14]. Amakiri et al. [36] compared the expression of nerve endings and axons in the neck region in three domestic ruminants (bulls, sheep, and goats), describing no histological differences between the three species and that nerve endings expressing substance P (SP) are present. This suggests that SP-related nociceptors could minimize pain during slaughter by neck cutting without previous stunning. Since pain requires consciousness and cerebral activity, Sabow et al. [37] investigated the electroencephalographic (EEG) response associated with noxious stimuli by cutting the neck during the slaughter of 10 male Boer goats. The authors reported that the theta, alpha, beta, and delta waves significantly increased 90 s after neck cutting in anesthetized animals. The increase in brain activity during slaughter can be interpreted as the recognition of noxious stimuli and the activation of A delta and C fibers that will transmit the signal to the dorsal horn of the spinal cord [38,39].

The transduction and transmission of noxious inputs in White Fulani (zebu breed), through histological and histochemical analyses, showed the presence of unmyelinated C fiber in hairy areas or with thick skin [36]. According to these results, the high density of type C fibers would explain the higher threshold activation observed in this species, compared to myelinic A fibers delta [40,41]. Thus, soft tissue cutting during exsanguination is a stimulus that elicits a nociceptive response.

The increased brain activity observed in animals during slaughter is enough to comprehend that an animal without stunning or is poorly stunned can develop hyperalgesia. Gibson et al. [42,43] conducted simultaneous studies on calves stunned with a non-penetrative captive bolt gun and slaughtered by ventral–neck incision and observed that the incision increased EEG activity while stunning decreased cerebral–cortical activity. These results suggest that the lack of stunning sensitized nociceptors transducing and transmitting the noxious stimulus. In goats, when the stimuli are not modulated in the dorsal horn of the spinal cord by inhibitory interneurons, the input is projected by ascendent pathways (e.g., spinothalamic) to superior brain centers such as the thalamus and the somatosensory cortex, two regions required for the conscious perception of pain [44].

In water buffaloes, the thickness of their skin and the short neck makes it difficult to perform the neck cut during slaughtering without stunning, thus increasing the pain. Hence, stunning can prevent hyperalgesia and sensitization by inactivating cerebral regions critical for pain recognition and inducing unconsciousness. Gibson et al. [15] evaluated the effect without stunning on the collapsing time in 755 Belgian Blue bovine. The cut at the neck was performed in two different regions: (1) the conventional low-cut, performed in front of the arytenoid cartilage without including the cricoid, and (2) the high-neck cut, below the level of the thyroid cartilage. The collapsing time in the low-cut group was 5.4 ± 1.1 s higher than the high-neck-cut group. This finding is relevant because the time when the animal remains conscious can initiate a cortical response together with its physiological consequences. Among said consequences, the activation of adrenergic fiber at the hypothalamus, amygdala, and hippocampus represents the release of catecholamines that consequently increase the activity of the sympathetic nervous system (SNS), increasing heart rate, blood pressure, and changes in the respiratory pattern [2,29].

**Figure 2:**
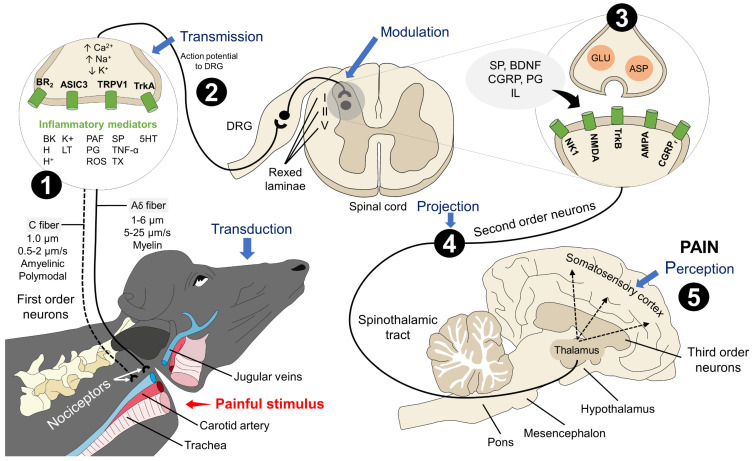
Nociceptive pathway activation during bleeding without previous stunning. Processes such as neck cut in conscious water buffalo imply the activation of the five stages of pain recognition. 1. After tissue injury at the neck, nociceptors detect and transduce the noxious stimuli into an electrical signal due to activating receptors and pro-inflammatory mediators (e.g., BK, H^+^, PG, PAF, SP, among others). 2. The transmission of this signal to the spinal cord is through Aδ and C fibers to synapse in the dorsal root ganglion (DRG). 3. In the spinal cord, modulation requires the interaction of several neurotransmitters (e.g., SP, BDNF, CGRP, PG, IL) and two main central receptors (AMPA and NMDA). 4. When excitatory neurotransmitters participate, the stimulus is projected to the thalamus (via the spinothalamic tract), where pain recognition starts. 5. Pain perception is achieved through the neuronal connections between the thalamus and the somatosensory cortex, triggering the physiological and behavioral responses observed in conscious animals. 5-HT: serotonin; AMPA: α-amino-3-hydroxy-5-methyl-4-isoxazolepropionic acid; ASIC3: acid-sensing ion channel 3; ASP: aspartate; BDNF: brain-derived neurotrophic factor; BK: bradykinin; BR2: bradykinin receptor 2; CGRP: calcitonin gen-related peptide; CGRPr: calcitonin gen-related peptide receptor; GLU: glutamate; H^+^: hydrogen; H: histamine; IL: interleukin; K^+^: potassium; LT: leukotrienes; NK1: neurokinin receptor 1; NMDA: N-methyl-D-aspartate receptor; PAF: platelet-activating factor; PG: prostaglandin; ROS: reactive oxygen species; SP: substance O; TNF-α: tumor necrosis factors alpha; TRPV1: transient potential receptor vanilloid 1; TrkA: tyrosine kinase receptor A; TrkB: tyrosine kinase receptor B; TX: thromboxane.

Figure 2 shows the neurobiological process of nociception, and its five phases (transduction, transmission, modulation, projection, and perception). The prevention of the last two phases is the objective of the stunning that involves the destruction of central nervous system neurons. The studies using physiological, endocrine, and EEG parameters explain why an inappropriate stunning method is associated with acute pain perception.

### 3.2. Peripheral and Central Sensitization during and before Water Buffalo Slaughter

The activation of nociceptive pathways during slaughter can be associated with sensitization when ineffective stunning causes the repetitive activation of peripheral/central nociceptors in response to inflammatory mediators such as cytokines, leukotrienes, PG, PC, SP, BK, histamine, 5-HT, H^+^, and K^+^ ions [2,45,46]. The constant activation of receptors in nociceptive fibers reduces the activation threshold, facilitating the creation of potential actions that will be projected to the brain in a phenomenon called primary or secondary hyperalgesia [47].

**Figure 3:**
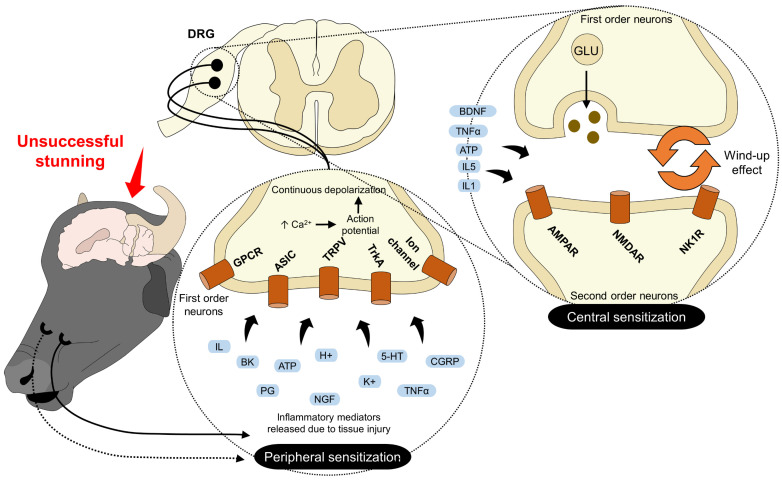
Peripheral and central sensitization during slaughter. When animals are conscious during slaughter, two events related to pain processing can be present. Peripheral sensitization results from the constant activation of receptors located in first-order neurons. GPCR, ASIC, TRPV, TrkA, and ion channels interact with the mediators released after tissue injury. These neurons’ constant activation and depolarization cause an accumulation of action potentials that keep transmitting pain signals to the spinal cord. Central sensitization refers to a similar process that occurs in second-order neurons at the DRG of the spinal cord. Here, receptors such as AMPAR, NMDAR, and NK1R lower their activation threshold due to the accumulation of potential actions, resulting in a wind-up effect where AMPAR and NMDAR can activate each other, maintaining a persistent sending of the painful signal. Both types of sensitization can be prevented by inducing unconsciousness in the animals. 5-HT: serotonin; AMPAR: α-amino-3-hydroxy-5-methyl-4-isoxazolepropionic acid; ASIC: acid-sensing ion channel; BDNF: brain-derived neurotrophic factor; BK: bradykinin; CGRP: calcitonin gen-related peptide; CGRPr: calcitonin gen-related peptide receptor; DRG: dorsal root ganglion; GLU: glutamate; H^+^: hydrogen; H: histamine; IL: interleukin; K^+^: potassium; LT: leukotrienes; NK1R: neurokinin receptor 1; NMDAR: N-methyl-D-aspartate receptor; PG: prostaglandin; ROS: reactive oxygen species; SP: substance O; TNF-α: tumor necrosis factor alpha; TRPV: transient potential receptor vanilloid 1; TrkA: tyrosine kinase receptor A.

With this reasoning, it is possible to suggest that a more significant tissue lesion might cause a greater inflammatory phenomenon. Although the inflammation occurring during bleeding without stunning may be the leading cause of hyperalgesia and peripheral sensitization (Figure 3), previous pathologies such as fractures and muscle or superficial tissue injuries generated during transport or handling can also contribute. During transport to the slaughterhouse and herding the animals to the stunning box, stressors such as screaming, back-slapping, or using electric drivers can induce injuries, pain, fear, and anxiety [48,49]. In this sense, Kumar et al. [50] evaluated the physiological and EEG response in 12 Boer goats exposed to other animals at the slaughter. It was determined that exposure to noise and shouting during these events increased beta and theta waves and the median frequency on EEG. This reaction was accompanied by a significant increase in blood glucose without effects on heart rate. These findings demonstrate that fear and anxiety during slaughter may influence the perception of nociception [29,51].

Likewise, according to José-Peréz et al. [9], the water buffalo is susceptible to trauma during mobilization due to inadequate vehicle design, vehicle size, stocking density, driving conditions, rough handling during loading, and road conditions. In this context, Kober et al. [52] conducted a study comparing the incidence of injuries between domestic cattle and water buffaloes under similar transport conditions. The authors reported a high incidence of lesions in both species, with a frequency of abrasions, lacerations, bleeding, and scarification in cattle of 73%, 45%, 4%, and 83%, respectively. In comparison, it was 71%, 9%, 23%, and 41% in buffaloes, respectively. This could demonstrate that the incidence of lesions would be comparable between cattle and water buffaloes; however, this would not diminish the importance of the fact that inflammation can affect the neuroplasticity of sensory fibers and their ability to transmit nociceptive stimuli. Thus, chronic inflammatory lesions affect the neuroplasticity of sensory fibers, not only in the peripheral fibers but in second-order neurons located in the spinal cord. Here, chemical mediators such as histamine, 5-HT, BK, and interleukins (IL-1 and IL-10) stimulate N-methyl-D-aspartate (NMDA) receptors, which reduce the action potential in excitatory neurons, leading to central sensitization [53]. These phenomena, together with the tissue damage of the neck cut, add harmful inputs to the neurobiological process of pain that can lead to signs of hyperalgesia [28].

In this regard, Mota Rojas et al. [2,3] mention that peripheral and central sensitization prior to slaughter could potentially increase nociception perception during slaughter [14,54]. Therefore, central and peripheral sensitization phenomena might contribute to nociception. Moreover, the exposure of the animals to previous injuries during transport and neck cutting triggers signs of hyperalgesia during slaughter.

## 4. Stunning Methods for Water Buffaloes

Rapid and effective stunning methods to achieve the unconsciousness of the water buffalo are required to ensure welfare during slaughter. Mechanical and, to lesser extent, electrical methods are the main stunning techniques applied to bovine species. In the former, a brain concussion is induced by the impact of a penetrative captive bolt, non-penetrative or free projectiles on the buffalo’s skull. For electrical methods, electric currents are applied to induce a generalized epileptiform activity in the brain, which can combine with generating the fibrillation of the heart or cardiac arrest. Ineffective stunning has been associated with the persistence of consciousness during sticking, hoisting, and bleeding [3,22].

### Penetrating Captive Bolt Pistol

**Figure 4:**
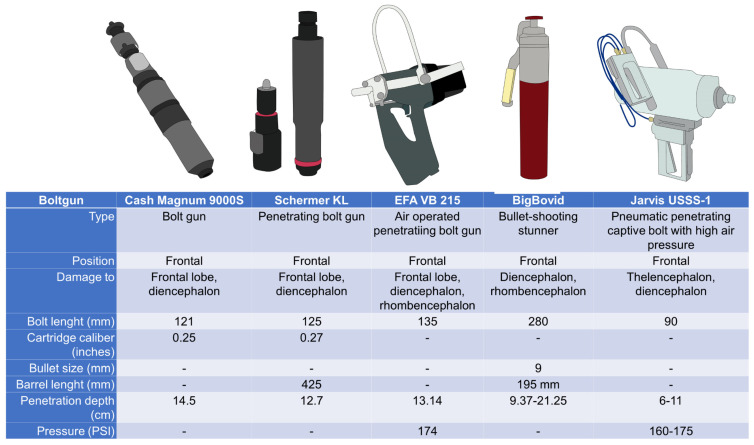
Technical characteristics of some devices used and proposed for stunning water buffaloes.

Deep unconsciousness is achieved by damage to brain regions associated with consciousness, such as the brainstem and thalamus. Penetrating captive bolt pistols cause the destruction of the mentioned cerebral structures, in contrast to non-penetrative devices that can cause extensive hemorrhage around the cerebellum but do not cause macroscopic lesions in the brainstem [55]. Thus, effective stunning must consider anatomical features in water buffaloes regarding the frontal and nasal sinuses and frontal and parietal bones. Considering these factors, various devices have been proposed to achieve effective stunning in water buffalo species in which only penetrating methods have been tested. This might be because non-penetrating captive bolt guns do not produce physical brain damage. Therefore, these devices should be avoided due to the higher incidence of ineffective stunning and the shorter duration of unconsciousness compared to penetrative captive bolt stunning. Figure 4 shows some examples of their technical characteristics described in studies by Schwenk et al. [19], Gascho et al. [56], and Molnar-Fernández et al. [21]. For example, Gregory et al. [4] proposed detonating captive bolt pistols in an occipital position to induce damage to the thalamus. When detonating penetrating captive bolt pistols in the frontal position, the required minimum depth is 143 mm (with maximum values of 172 mm). In contrast, the required average depth in the occipital region is 105 mm. Although an occipital position is recommended in water buffaloes, this change in the methodology might be impractical at a commercial level [19], and some countries have banned this type of stunning in *Bos* cattle due to the risk of sectioning the spinal cord or cerebellum, causing paralysis without loss of consciousness.

Some authors suggest modifications in the stunning frontal procedure. Molnar-Fernández et al. [21] studied conventional pneumatic penetrating captive bolts with high air pressure (PPCB) using 352 buffaloes and 168 post-mortem heads to determine the most effective anatomical site for stunning. The detonation was applied 8 cm dorsally above the middle of the forehead and 2 cm lateral, avoiding the midline. An effectiveness of 95% was obtained at the first shot, with animals collapsing and having the absence of corneal and palpebral reflexes, in addition to the absence of rhythmic breathing. The authors concluded an effective stunning technique with a PPCB at pressures of 1379–1516.8 KPa (200–220 pounds per square inch (psi)).

Modifications have also been implemented on captive bolt pistols, increasing propellant charges and their bolt length by 50%, ranging from 120 mm in conventional cattle to 180 mm for water buffaloes to address the anatomical differences [19]. Researchers such as Gascho et al. [56] implemented a bolt and free projectile stunning method (BigBovid) on the frontal bone to achieve desensitization and a loss of consciousness for heavy bovines. Using 0.38 SPL FMJ-TC and 0.357 MAG FTX^®^ bullets in a 280 mm of length device, it was found that 0.357 MAG FTX^®^ bullets correctly penetrated the thalamus and brainstem because these bullets fragment immediately, leaving various scattered fragments in the brain tissue and remaining inside the cranial cavity.

## 5. Methods to Evaluate the Quality of Unconsciousness, Return-to-Sensibility Signs, and Death

**Figure 5:**
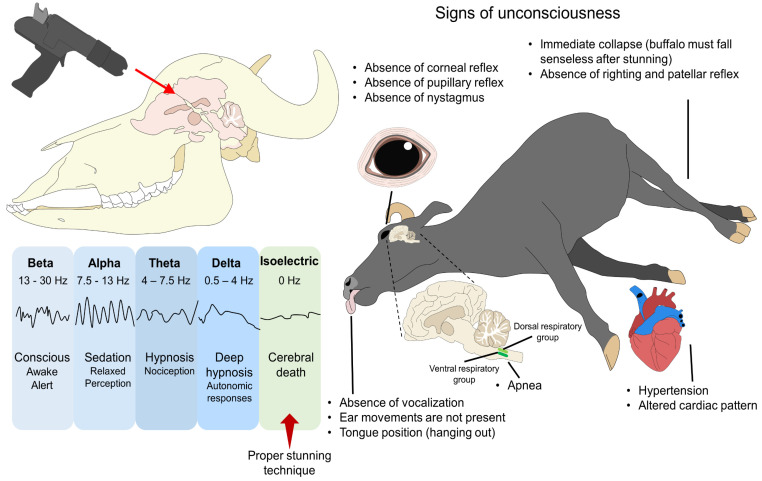
Signs to evaluate unconsciousness. The objective of stunning is to induce an immediate state of unconsciousness and cerebral death, represented by an isoelectric trace. Several signs can be used to evaluate the depth of unconsciousness. For example, correctly stunned animals must have an absence of corneal, pupillary, and absent nystagmus. Immediately after stunning, the animal must collapse and is not able to respond to nociceptive stimulus. There might be a tonic seizure and padding movements, but the animal will not try to incorporate (righting reflex). The lack of vocalization—however, not all conscious animals vocalize—or ear movements and the tongue protruding out of the mouth (due to muscle tone loss) must also be accompanied by apnea and alteration in the cardiac pattern to guarantee an appropriate level of unconsciousness.

To ensure a humane slaughter (without pain), the state of unconsciousness or return-to-sensibility signs must be assessed through behavioral observation and physiological reflexes, as presented in Figure 5 [57]. Since stunning implicates the damage to cerebral hemispheres, thalamic structures, or the ascending reticular activation system, all the reflexes mediated by brainstem mechanisms must be absent to assess the cessation of brain function indirectly [58,59]. When not performed correctly, ineffective and reversible stunning can lead to a lack of unconsciousness or the recovery of consciousness before death. For this reason, it is necessary to recognize specific signs that could indicate consciousness and, consequently, pain [3,60].

**Figure 6:**
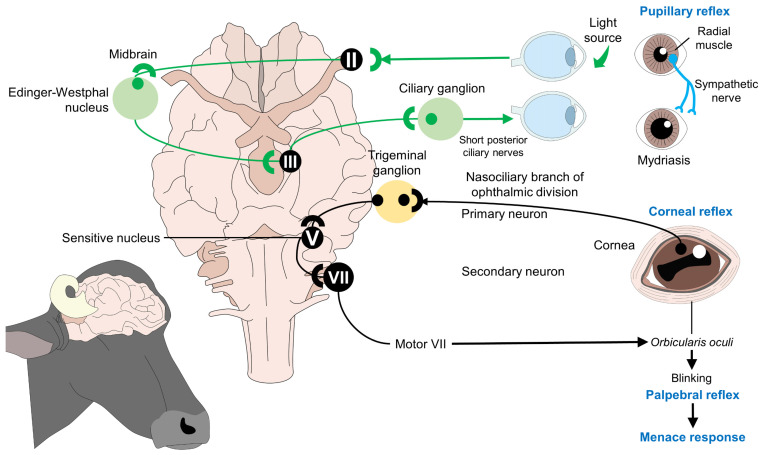
Cranial nerves and reflexes are associated with consciousness. To elicit reflexes such as the pupillary, corneal, palpebral, and menace response, the integrity and functionality of the brainstem and its motor projections must be present. Therefore, in stunned animals, the absence of these reflexes indicates the degree of damage to cerebral structures and, consequently, the correct application of the stunning technique. II: Optic nerve; III: oculomotor nerve; V: trigeminal nerve; VII: facial nerve.

Gregory et al. [23] mention that reflexes related to consciousness must be absent immediately after stunning to determine the technique’s efficacy before slaughter [13]. Among the indicators to evaluate consciousness, attempts to regain posture and collapse failure, eyelid and corneal reflexes with blinking and full eyeball rotation, head raising, and respiratory rhythm, are included. Figure 6 shows that this is due to the innervation of the eye and its interaction with cranial nerves, which emerge from the brainstem and control critical reflexes associated with consciousness. The trigeminal (V) and facial (VII) nerves control the palpebral, corneal, and menace reflexes. In these three reflexes, the ophthalmic division of the V nerve synapses with its respective sensitive nucleus and then with the motor branch of the VII nerve that directly innervates the orbicularis oculi muscle. The integration of these pathways results in blinking, a response that is absent in unconscious individuals. However, the presence or absence of blinking per se cannot be considered an indicator of unconsciousness.

On the other hand, the pupillary reflex, controlled by the optic (II) and oculomotor (III) cranial nerve, is elicited by a light stimulus. In a conscious animal, the action of the oculomotor on the pupillary sphincter would cause pupil contraction due to the interconnections and functionality of the brainstem. However, during stunning, this reflex should be absent after damage to the cerebral parenchyma [58,61,62,63,64]. Terlouw et al. [58] mentioned that apart from the cited indicators, correctly stunned animals cannot respond to painful stimuli and do not vocalize after stunning [64].

A study by Terlouw et al. [65] evaluated the origin of the frequency of movements in cattle during slaughter after penetrative captive bolt stunning and neck cutting. They observed that the motor incoordination originating in the brainstem and spinal cord could continue for up to 3 min in stunned and unconscious cattle after bleeding. The presence and frequency of movements was not influenced by the section of the spinal cord. These authors concluded that factors such as the location of the shot and the initial efficiency of the bleeding in the neck cutting are the main factors influencing the frequency of movements and the influence of the type of bleeding.

Atkinson et al. [63] developed a stun system to categorize the quality of the technique in 998 cattle (585 bulls, 306 cows, 58 steers, and 49 calves) in commercial abattoirs. The authors considered shot accuracy, the stun-to-stick interval times, the repetition of the shots, and the signs of a return to consciousness, classifying the quality of the stunning on a scale from 3 to 1 (from highest to lowest risk for animal welfare). The scores were assigned as follows: 3: the absence of animal collapse, rhythmic breathing, responses to pain, blinking, vocalizations, and corneal reflexes; 2: a complete rotation of the eyeball and the presence of nystagmus; 1: gasping, groaning, tongue retained in the mouth, ears not pointing downwards, and reactions to sticking, as well as the absence of tonic/clonic phase and partial eye rotation. The results showed that 12.5% of animals had quality stun rates of 3 and 2, suggesting that higher caliber weapons (>0.22 caliber) are needed, and the implementation of head restraints would help to improve the technique and regular servicing of the weapons [63].

### Ocular Signs of Consciousness during Slaughter

One of the proposed signs to assess the animal’s level of consciousness is the assessment of pupillary reactivity, palpebral reflex, corneal reflex, and nystagmus presence. The brainstem modulates these signs and their connection to the cerebral cortex [66]. For example, the palpebral and corneal reflexes are assessed by touching the medial canthus of the eye with the finger or paint brush, causing the lid to close. The absence of this reflex indicates the lack of functionality of the brainstem [58]. However, the use of these indicators may be limited, and their evaluation must be performed together with other indicators. Comin et al. [67] mentioned that the stunning method could compromise the corneal and palpebral reflexes’ function, as observed with the use of captive bolt stunning and probably electrical stunning.

Reflexes, together with the suggested complementary indicator, are the evaluation of the pupillary response, which is related to the activity of the pupillary sphincter and iris dilator muscles, innervated by cranial nerves II (optic nerve) and III (oculomotor nerve) [68]. The pupillary response is evaluated by stimulating the pupil with direct light, generating its contraction due to the participation of the retina, the optic nerve, and the accessory oculomotor nucleus (Edinger–Westphal) in the midbrain, with outputs mediated through the parasympathetic endings of the oculomotor nerve. Since the pupillary reflex requires the connection of the brainstem with the spinal cord and sympathetic fibers, the absent reaction in the stunned animals indicates a correct state of unconsciousness [69,70].

Due to the complex functional network of the pupillary reflex, the absence of this response indicates unconsciousness, which can be associated with the degree of nociception due to the activation of the SNS that can lead to pupillary dilation. Figure 6 shows this reaction and its possible association as an ocular indicator to assess the state of consciousness. Therefore, signs of consciousness and voluntary movements possibly indicate the existing communication pathways between the cerebral cortex and the brainstem when stunning is not applied correctly.

## 6. Future Research Aimed to Improve Stunning Methods in Water Buffaloes

The perspectives regarding stunning methods, sticking procedure, and their relation to pain in water buffaloes include studies addressing the distribution of the nociceptors in the tissues surrounding the neck and the activation and expression levels of ion channels in the species. Regarding the interaction of pro-inflammatory mediators and their action on specific receptors, it is essential to determine objective methods to assess pain-related biomarkers, autonomous responses, and brain activity in water buffaloes. For example, while EEG has been used in the *Bos* genus cattle to determine the effect of stunning or neck-cutting methods [42,71], there is no available information for water buffaloes.

Likewise, a potential field of research is additional comparative and anatomical studies between conventional cattle and water buffaloes to improve the current management of animals during slaughtering. Moreover, this could also be applied to other large ungulate species with partly similar skull structures in whom conventional stunning techniques might be less efficient. In addition, it is important to recognize that pain is a multimodal process that is not solely influenced by the technique or device, but by stressors such as fear, anxiety, inadequate handling, and overcrowding, as observed in small ruminants [50]. Moreover, phenomena such as stress-induced analgesia is known as an adaptive response to survival, as reported in laboratory rodents [72]. Its study on water buffaloes and other ruminant species is another topic to address in the future.

It is important to highlight the limited information regarding the adequate stunning technique and gun position in water buffaloes. The selection for the occipital region (poll) or the positioning at 2 cm of the central line are suggested to achieve the damage to cerebral structures involved in consciousness [4,54]. However, precautions should be taken since damage to the spinal cord, without a loss of consciousness, has been observed. Therefore, a pneumatic penetrating captive bolt with high air pressure might be more suitable for water buffaloes [21].

Finally, the consequences of an inadequate stunning or bleeding technique, such as false aneurysms, need to be investigated in water buffaloes. Knowing that false aneurysms can delay the loss of consciousness in non-stunned buffaloes and increase pain perception, evaluating methods of cutting to minimize aneurysms and where bleeding is performed without prior stunning could show the importance of providing a quality of death in buffaloes [13,73].

## 7. Conclusions

The assessment of appropriate stunning methods for water buffaloes is a topic that requires the consideration of several biological, anatomical, technical, and comparative aspects. Although *Bubalus bubalis* and *Bos taurus* are bovines, there are significant differences in the skull structure that make stunning devices for cattle inappropriate for water buffaloes. The water buffalo’s hide thickness and width of the frontal sinus are larger than in conventional cattle. This means that conventional sites for stunning in cattle cannot be performed in water buffaloes, and alternative devices are required.

The consequences of an ineffective or the lack of a stunning technique need to be considered. For example, acute pain, false aneurysms, blood aspiration, and return-to-sensibility signs are events that need to be prevented to promote the welfare and quality of death in water buffaloes.

This literature report modified stunning devices with captive bolt guns to guarantee thalamus and brainstem damage. Very high-pressure pneumatic equipment is recommended for smaller animals, while bullet guns are the most appropriate for larger animals. However, non-penetrating captive bolt devices should not be used on water buffaloes because the literature shows that they are less effective than in cattle. Further research is required to establish these strategies into official guidelines for water-buffalo-slaughter processes.
